# Effects of a Single Sub-Anesthetic Dose of Ketamine in Tobacco Use Disorder: An Active-Placebo, Randomized Crossover Study

**DOI:** 10.3390/brainsci16050496

**Published:** 2026-04-30

**Authors:** Nathan R. Luzum, Marcia H. McCall, Charlotte Talley Boyd, Heather Columbano, Edward Ip, Santiago Saldana, Alison H. Oliveto, Merideth Addicott

**Affiliations:** 1 Department of Psychiatry and Behavioral Medicine, Wake Forest University School of Medicine, Winston-Salem, NC 27101, USA; nathan.luzum@wfusm.edu (N.R.L.);; 2 Department of Family and Community Medicine, Wake Forest University School of Medicine, Winston-Salem, NC 27101, USA; 3 Department of Anesthesiology, Wake Forest University School of Medicine, Winston-Salem, NC 27101, USA; 4 Department of Biostatistics and Data Science, Wake Forest University School of Medicine, Winston-Salem, NC 27101, USA; 5 Department of Psychiatry, University of Arkansas for Medical Sciences, Little Rock, AR 72205, USA; 6 Department of Translational Neuroscience, Wake Forest University School of Medicine, Winston-Salem, NC 27101, USA

**Keywords:** ketamine, tobacco use disorder, cigarette smoking, substance use disorders, nicotine

## Abstract

**Highlights:**

**What are the main findings?**

- Ketamine resulted in a non-significant reduction in short-term smoking during a brief period of requested abstinence, but not in ad lib smoking over a 1-week period.
- Some participants reported ketamine briefly reduced craving for cigarettes and anxiety.

**What are the implications of the main findings?**

- Among people not interested in quitting, ketamine has little effect on smoking behavior.
- Ketamine could theoretically support a quit attempt by improving mood and alleviating distress, but further investigation is required to support potential efficacy.

**Abstract:**

**Background/Objectives:** A sub-anesthetic dose of ketamine has shown promise in reducing craving, withdrawal symptoms, and use of drugs such as alcohol, cocaine, and opioids among individuals with substance use disorders. Ketamine’s therapeutic potential for tobacco use is unknown. Here, we investigated a single sub-anesthetic dose among adults with tobacco use disorder who were not interested in changing their smoking behavior. **Methods:** Utilizing a randomized, within-subject crossover, double-blinded, counter-balanced, midazolam-controlled design, participants (*n* = 18) received a 0.71 mg/kg infusion of ketamine and a 0.025 mg/kg infusion of midazolam (i.e., active placebo) at least two weeks apart. Participants were asked to abstain from smoking after the infusions until the post-infusion sessions, 1 day following infusion, where participants completed measures of smoking behavior, craving, and withdrawal symptoms. Participants continued to record their smoking behavior over the 7 days following infusion. Participants also completed a semi-structured qualitative interview regarding their experiences. **Results:** Compared to midazolam, ketamine infusion led to a non-significant reduction (*p* = 0.10, η_p_^2^ = 0.153) in the number of cigarettes smoked during the requested abstinence period. Following this period, there were no significant differences in ad lib smoking. Ketamine showed no effect on craving or withdrawal symptoms. Participants reported more intense psychological experiences following ketamine infusion (*p* < 0.001, η_p_^2^ = 0.830) and about half reported it felt easier to abstain from smoking after the ketamine infusion. **Conclusions:** While well tolerated, these findings suggest ketamine has little to no direct effect on quantitative measures of cigarette smoking, craving, or withdrawal. However, the qualitative measures suggest ketamine improves mood and reduces craving in some individuals for several days. Future studies should investigate whether ketamine can indirectly support smoking cessation among individuals with comorbid psychiatric indications for ketamine treatment.

## 1. Introduction

Habitual tobacco use is associated with a number of individual health risks, and a massive strain on the healthcare system and society at large. According to the 2014 Surgeon General’s report, there are over 480,000 deaths attributable to cigarette smoking annually in the United States alone [1]. In the year 2020, healthcare expenditures attributable to smoking reached $18.9 billion in the U.S. [2]. Fortunately, smoking cessation has been shown to reduce this risk of excess morbidity and mortality in former smokers [3]. Therefore, it is imperative for clinicians and researchers to develop and utilize effective interventions to aid in cessation.

Beyond nicotine replacement therapy, there are currently just two Food and Drug Administration (FDA) approved pharmacologic interventions for smoking cessation: varenicline and bupropion [3]. In a large, multicenter, randomized controlled trial, Anthenelli et al. found six-month smoking cessation rates of 25.5%, 18.8%, and 18.5% for varenicline, bupropion, and nicotine replacement, respectively [4]. While each of these are more effective than placebo (10.5% six-month abstinence), the majority of smokers are unable to quit despite using currently available treatments, highlighting the need for novel interventions.

Ketamine is an N-methyl-D-aspartate (NMDA) receptor antagonist that has traditionally been utilized as an anesthetic agent, but more recently has gained attention for its potential for use for various psychiatric conditions, including mood and substance use disorders. The glutamate homeostasis hypothesis of addiction provides one possible explanation for the potential efficacy of ketamine in substance use disorders. This hypothesis, derived from research in animal models, postulates that the diminished ability of individuals to control drug seeking and consumption in substance use disorders stems from a dysregulation in glutamate transmission at synapses connecting the prefrontal cortex to the nucleus accumbens [5]. This dysregulation may involve long-term depression of glutamate-mediated synaptic connections that mediate non-drug reinforcement of behavior, while potentiating connections that influence drug-seeking and drug cue reactivity. Therefore, it has been suggested that drugs that modulate glutamate activity, such as ketamine, may aid in restoring this homeostasis, and could be useful in treating addiction [5,6]. In support of this hypothesis, ketamine infusion was associated with a significant reduction in nicotine self-administration in a rat model [7].

There is also evidence that ketamine enhances neuroplasticity, which is another leading hypothesis in explaining its potential benefit in mood and substance use disorders [8,9]. Recent evidence suggests that substance use disorders result in part due to maladaptive neuroplastic changes in various brain regions, and interventions that enhance neuroplasticity have the potential to counteract or reverse this [10]. Ketamine has been described as a “psychoplastogen,” which is a group of drugs that induce robust changes in neuronal growth, and includes psychedelic compounds such as lysergic acid diethylamide (LSD) and psilocin [8]. This increase in neuronal growth was found to last up to 2 weeks in a rodent model after a single administration of ketamine [11]. While the specific mechanisms underlying this plasticity have not been definitively established, there is evidence to suggest that changes in gene expression, specifically increases in brain-derived neurotrophic factor (BDNF) and mechanistic target of rapamycin (mTOR) activation, play a role, with some studies finding a resultant increase in dendritic spine density in the prefrontal cortex and hippocampus [8,9,12].

A 2021 systematic review identified 14 original studies that have examined the effects of ketamine in substance use disorders [13]. These studies have indicated benefits of ketamine in reducing substance use among individuals with alcohol use disorder [14,15], cocaine use disorder [16,17,18] and opioid use disorder [19], to varying degrees. To date, the only study of ketamine in human subjects with tobacco use disorder (TUD) was a pilot study performed by our group, which utilized a single-blind and inactive placebo design, and failed to identify significant effects on cigarette smoking, craving, or withdrawal symptoms [20]. The purpose of this study is to expand on this work in a randomized, within-subject crossover, double-blind, active-control trial to better characterize the potential benefits of a single ketamine infusion in non-treatment-seeking participants with tobacco use disorder.

## 2. Materials and Methods

### 2.1. Participants

Adults aged 18 to 65 who smoke combustible cigarettes were recruited from the Winston-Salem, NC area, by social media and word of mouth. Inclusion criteria included smoking at least 5 cigarettes per day, smoking for at least 2 years, positive urine cotinine and afternoon expired breath carbon monoxide (CO) concentrations ≥5 parts per million (ppm). Additionally, participants were only included if they had no plans to change their smoking behavior in the next 60 days. Participants were excluded if they had certain chronic medical conditions, psychiatric diagnoses of schizophrenia or bipolar I disorder, any current psychiatric illness causing significant distress, moderate to severe substance use disorder (other than tobacco/nicotine) per Diagnostic and Statistical Manual-5 (DSM-5) criteria, a positive urine drug screen for certain drugs of abuse, or any history of nonmedical use of ketamine or benzodiazepines. See Appendix A for complete details. Screening involved the collection of data on baseline cigarette consumption, including DSM-5 TUD and Fagerström Test for Nicotine Dependence scores. Participants were compensated up to $800 for completing all aspects of the study. All participants gave written informed consent, and this study was approved by the Wake Forest University School of Medicine Institutional Review Board (IRB00081012). This trial was pre-registered at Clinicaltrials.gov (NCT05505630). This study was part of a parent protocol that included magnetic resonance imaging (MRI); those results will be reported elsewhere.

### 2.2. Experimental Design

**Figure 1:**
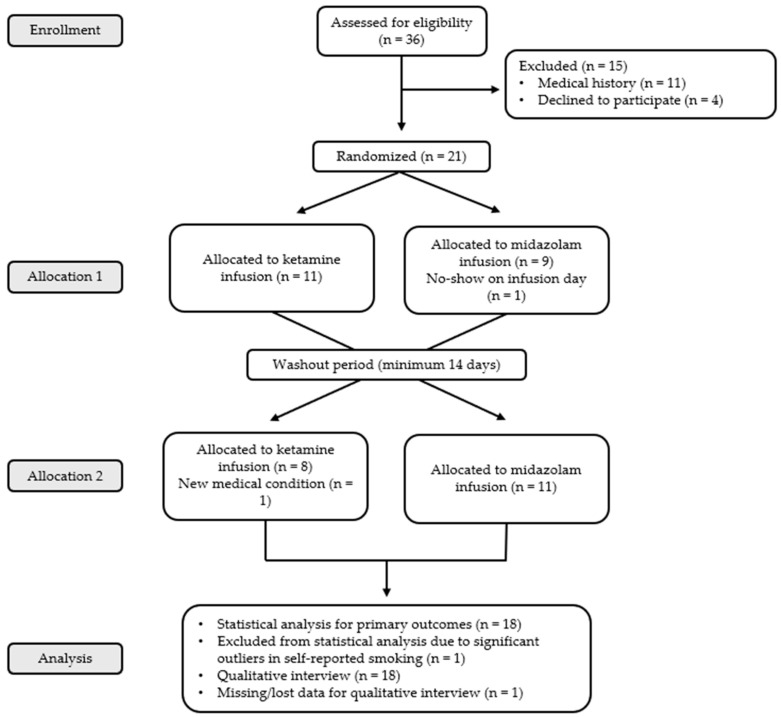
Consolidated Standards of Reporting Trials (CONSORT) diagram depicting the experimental design utilized in this randomized controlled trial.

This study utilized a within-subject crossover design with double-blind drug administration (Figure 1). Participants either received ketamine during the first infusion followed by midazolam, or received midazolam during the first infusion followed by ketamine. Midazolam was used as a psychoactive control to help maintain the double-blind. Participants then completed post-infusion study visits 1–2 days after each infusion to evaluate withdrawal, craving, drug effects, and mood symptoms. The second infusion session was scheduled for at least 2 weeks following the first infusion to allow for washout of any residual drug effects.

Beginning on each infusion day, participants kept a daily smoking diary for 7 days. Participants were allowed to smoke prior to each infusion and were then instructed to abstain from all tobacco or nicotine use until the post-infusion study session to evaluate withdrawal symptoms. Breath CO was obtained at each infusion and post-infusion session. Breath CO concentrations of ≤4 ppm were used to verify 24 h smoking abstinence [21]. Participants were not excluded from the study if they were not able to achieve this period of abstinence.

### 2.3. Infusion Protocol

On the day of infusion, participants were told they may receive ketamine, midazolam, dexmedetomidine, or placebo to minimize drug expectations (only ketamine or midazolam were actually administered). Drug assignment was double-blinded to subjects and study staff. On the ketamine infusion day, subjects received an infusion of ketamine at a dose of 0.71 mg/kg, modeled after Dakwar et al. [17]. A bolus dose of 0.11 mg/kg ketamine in 15.5 mL of normal saline (NS) was administered over 2 min to obtain a potent sub-anesthetic level of ketamine at the start of the infusion. Then, 0.6 mg/kg of ketamine in 84.5 mL NS was administered in a 50 min slow-drip infusion.

On the midazolam infusion day, subjects received an infusion of midazolam (0.025 mg/kg) in 100 mL of NS. A bolus dose of 15.5 mL NS was administered over 2 min to match the ketamine infusion procedure. Then, a 0.025 mg/kg dose in 84.5 mL of NS was administered in a 50 min slow-drip infusion. On both infusion days, subjects were offered an optional dose of ondansetron prior to infusion to prevent nausea/vomiting.

Infusions were conducted at the Brookstown Pain Clinic in Winston-Salem, North Carolina. The study doctor and nurse administered the medication, and the study coordinator remained at the participants’ bedside throughout the session. All participants were monitored according to the American Society of Anesthesiology Standards for Basic Anesthetic Monitoring during infusion of study drugs, including continuous assessment (i.e., every 5 min) of pulse oximetry, electrocardiography, and intermittent non-invasive oscillometric blood pressure measurement. These measures were documented every 15 min for the first hour (i.e., during the infusion), then again after 1 more hour. Participants were provided a ride service to and from infusion sessions.

### 2.4. Outcome Measures

#### 2.4.1. Safety, Tolerability, and Test of Drug Blind

Perceptual alterations during the infusion of ketamine and midazolam were measured using the Clinician Administered Dissociated States Scale (CADSS) [22]. The Drug Effects Questionnaire was utilized to quantify positive and negative drug effects (e.g., “Did you feel high?”). During the post-infusion study session, the Side Effects Questionnaire was administered to evaluate side effects associated with the infusion (e.g., nausea, headache, feeling faint, blurred vision, etc.). The 21-item Positive and Negative Affect Schedule (PANAS) was administered to measure current emotional state for positive and negative moods [23]. To determine if participants perceived a mystical experience (e.g., “I realized the oneness of myself with all things”), the Hood Mysticism Scale (HMS) was administered following drug infusions [24]. This scale involves rating various experiences related to mysticism on a scale of −2 (definitely not true) to +2 (definitely true). Following the infusion, participants and study staff were also asked to indicate if they believed they had received ketamine, midazolam, dexmedetomidine, placebo, or if they were not sure.

#### 2.4.2. Cigarette Smoking, Craving and Withdrawal

Participants self-reported the number of cigarettes they consume on a daily basis at the screening session, and during the time between the infusion and the post-infusion study sessions. Daily cigarette consumption was recorded every day for 7 days starting on the infusion session via patient self-report using an electronic diary (lifedatacorp.com; Marion, IN, USA). This measure included cigarettes smoked on day 1 prior to the infusion and on day 2 after the post-infusion study session. The Tiffany Questionnaire of Smoking Urges-Brief (QSU) was utilized to quantify cigarette craving by measuring urges to smoke in response to positive or negative reinforcement [25]. The Minnesota Nicotine Withdrawal Scale (MNWS) was used to measure nicotine withdrawal symptoms [26].

#### 2.4.3. Qualitative Interviews

At the completion of study participation, the study coordinator conducted semi-structured interviews to gather qualitative feedback on participants’ experiences with the infusions. The interview was conducted after each participant completed all other aspects of the study and occurred at least 7 days after their second infusion. Each participant was asked to describe (1) their experiences during the infusions, (2) if they experienced any changes in thought patterns, behaviors, or habits after either infusion, and (3) if either infusion made it easier or more difficult to abstain from smoking for 24 h. Interview transcripts were imported and coded in ATLAS.ti version 24 software (Berlin, Germany; Lumivero, LLC). Two researchers independently coded the textual data and met to resolve any discrepancies. Code summaries were synthesized into themes and organized using principles of thematic analysis [27]. Specifically, themes were identified by using the following criteria: (1) prevalence, or frequency of an idea being shared; (2) strength, or emphasis given to a particular idea; and (3) valence, or relevance to the research context.

### 2.5. Statistical Analysis

Statistical analysis was conducted under an extended Generalized Linear Model framework. First, repeated-measures analysis of variance (ANOVA) tests evaluated within-subject drug and time effects utilizing SPSS v27 (Armonk, NY, USA; IBM Corp). Seven-day cigarette diaries were analyzed using a Generalized Estimating Equation (GEE). Diary data was missing from 9 entries across 6 participants. The GEE regression model utilized R-based program geeglm [28]. Two sources of within-subject correlation in GEE were identified: temporal correlation within a session (ketamine-first vs. midazolam-first) and correlation across sessions. A first-order autoregressive correlation structure was specified to handle the temporal correlation, whereas a robust estimator was applied to account for across-session correlation within the same subject. Side effect frequencies were analyzed using chi-square tests.

A two-sided test with significance level of 0.05 was utilized for all analyses. Due to the small sample size of this study, outcomes are reported in terms of effect size in addition to statistical significance; large effect sizes are noted (η_p_^2^ ≥ 0.14) for ANOVA tests, and regression coefficients with confidence intervals are reported for GEE.

## 3. Results

### 3.1. Participants

Twenty-two adult tobacco smokers were screened as eligible for participation in this study. Nineteen of these participants completed all aspects of the study. One of these participants was excluded from statistical analysis due to significant outlier values in self-reported cigarette consumption but was included in the qualitative interview report. The statistical analysis consisted of 18 adult tobacco smokers, with ages ranging from 26 to 64 years (Table 1). The interval between the infusion and post-infusion study session was two days on two occasions due to scheduling constraints related to MRI availability for the parent study (average time to follow up of 29.0 ± 6.2 h). No participants reported prior use of ketamine.

### 3.2. Safety, Tolerability, and Test of Drug Blind

**Figure 2:**
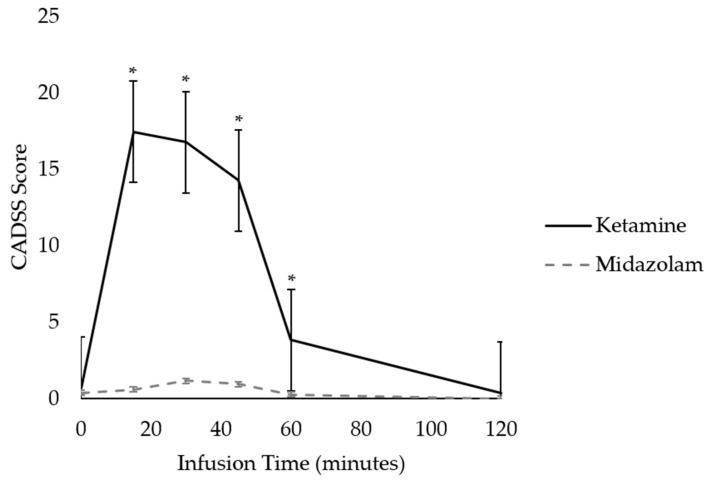
Clinician Administered Dissociated States Scale (CADSS) ratings during and after infusion of ketamine and midazolam. Infusions lasted 52 min. * *p* < 0.05.

There were no unexpected serious adverse effects during or after ketamine or midazolam infusion. One participant did have an episode of emesis during the ketamine infusion, with resolution of symptoms following administration of promethazine. On average, ketamine was associated with a slight, transient increase in blood pressure, heart rate, and oxygen saturation, while the opposite was true of midazolam (Appendix B). Participants experienced significantly greater CADSS dissociative effects during ketamine infusion compared to midazolam, which returned to near-baseline levels approximately one hour after infusion completion (Drug x Time Effect: F(5,13) = 3.33, *p* = 0.037, η_p_^2^ = 0.562). Follow-up Bonferroni-corrected *t*-tests revealed significant differences between ketamine and midazolam at timepoints 2 through 5 (*p*’s < 0.05) (Figure 2).

**Figure 3:**
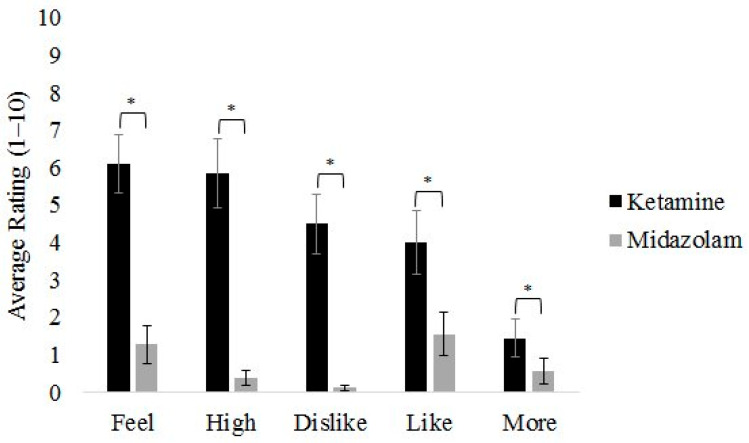
Responses on the Drug Effects Questionnaire following ketamine and midazolam. The five dimensions are (1) feeling any effects, (2) feeling a subjective high, (3) disliking the drug effect, (4) liking the drug effect, and (5) wanting more of the drug. * *p* < 0.05.

In the Drug Effects Questionnaire, ketamine was rated as having a significantly higher overall psychoactive effect (Drug Effect: F(5,13) = 16.44, *p* < 0.001, η_p_^2^ = 0.863), which was statistically significant with large effect sizes in all five domains: feeling a drug effect (F(1,17) = 21.24, *p* < 0.001, η_p_^2^ = 0.555), feeling a subjective high (F(1,17) = 31.89, *p* < 0.001, η_p_^2^ = 0.652), liking the drug effect (F(1,17) = 6.43, *p* = 0.021, η_p_^2^ = 0.274), disliking the drug effect (F(1,17) = 30.98, *p* < 0.001, η_p_^2^ = 0.646), and wanting more of the drug (F(1,17) = 5.06, *p* = 0.038, η_p_^2^ = 0.229) (Figure 3).

At the post-infusion study sessions, there were no significant differences in the severity or frequency of any of the side effects measured in the Side Effects Questionnaire. Following ketamine, the most commonly reported side effects were sleepiness (*n* = 8), dry mouth (*n* = 5), anxiety (*n* = 5) and headache (*n* = 5). Similarly, following midazolam, the most commonly reported side effects were sleepiness (*n* = 7), dry mouth (*n* = 3), anxiety (*n* = 6), and headache (*n* = 3). There were no differences in PANAS positive (Drug Effect: F(1,17) = 0.001, *p* = 0.97, η_p_^2^ = 0) or negative (Drug Effect: F(1,17) = 1.90, *p* = 0.19, η_p_^2^ = 0.101) measures of emotional state.

The infusion of ketamine was associated with a significantly less negative “mystical” experience on the total HMS (−3.8 ± 25.1) compared to midazolam (–27.6 ± 33.0) (Drug Effect: F(3,15) = 3.61, *p* = 0.038, η_p_^2^ = 0.420). That is, the average mystical experience following ketamine infusion was largely neutral (with a range of responses that included strong mystical experiences), whereas experiences with midazolam were consistently rated as non-mystical. This difference in drug effect was significant and with large effect sizes for introvertive (F(1,17) = 10.26, *p* = 0.005, η_p_^2^ = 0.376) and interpretative (F(1,17) = 5.39, *p* = 0.033, η_p_^2^ = 0.241), but not for extrovertive (F(1,17) = 2.38, *p* = 0.141, η_p_^2^ = 0.123), subscale dimensions of mysticism.

Seven of the participants correctly identified that they had received ketamine following its infusion (38.9%), compared to just one correct identification of midazolam following its infusion (5.6%). Twelve participants believed they had received a non-active placebo following midazolam infusion (66.7%). The study coordinator correctly identified 15 (83.3%) of the ketamine infusions and 17 (94.4%) of the midazolam infusions (Table 2).

### 3.3. Cigarette Smoking, Craving and Withdrawal

**Figure 4:**
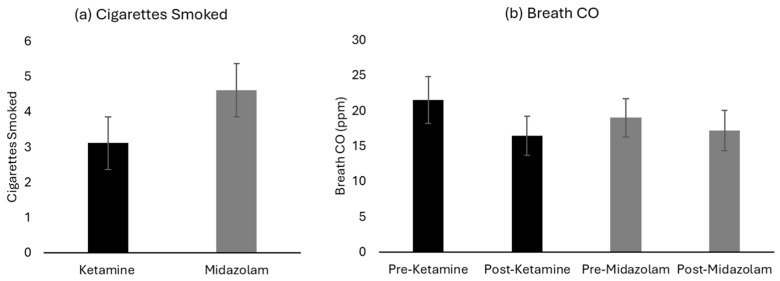
Primary outcomes at post-infusion follow-up visits. Participants were asked to abstain from smoking during this period of time (29.0 ± 6.2 h). (**a**) Cigarettes smoked in the period between infusion and post-infusion follow up by self-report. (**b**) Breath CO readings in ppm on the days of infusion and at post-infusion follow up.

In the requested smoking abstinence period (i.e., between infusion and post-infusion study sessions) participants averaged (mean ± standard deviation) 3.1 ± 3.2 cigarettes by self-report following infusion of ketamine, compared to 4.6 ± 4.7 cigarettes following infusion of midazolam (Figure 4). This difference was of large effect size but was not statistically significant (Drug Effect: F(1,17) = 3.07, *p* = 0.098, η_p_^2^ = 0.153). Breath CO concentrations non-significantly decreased more after ketamine (−5.11 ppm) than midazolam (−1.84 ppm), consistent with the trend in self-reported number of cigarettes (Drug x Time: F(1,17) = 1.52, *p* = 0.24, η_p_^2^ = 0.082). Only one participant was able to achieve complete abstinence from cigarette consumption between infusion and post-infusion study sessions (confirmed by breath CO ≤ 4 ppm), which was achieved after receiving both ketamine and midazolam. The duration of the period between infusion and post-infusion sessions varied slightly, but there was no significant difference between ketamine (30.0 ± 7.8 h) and midazolam (28.0 ± 4.1 h) infusions (Drug Effect: F(1,17) = 1.15, *p* = 0.30, η_p_^2^ = 0.063).

**Figure 5:**
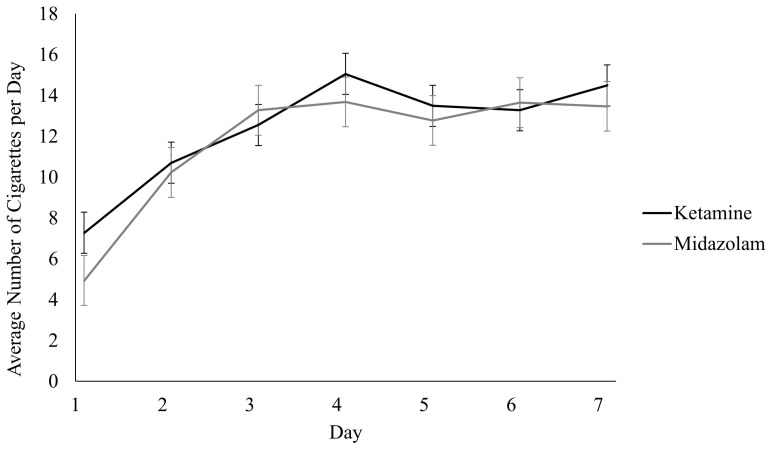
Total number of cigarettes smoked each day in the 7 days following the infusion of ketamine or midazolam. Participants were instructed to abstain from smoking between the infusion (day 1) and post-infusion study sessions (days 2–3: 29.0 ± 6.2 h).

There were no significant differences in cigarette consumption over the 7 days following ketamine and midazolam infusion (Drug x Time Effect: Β = 0.20, 95% CI: −0.48, 0.88, *p* = 0.56; Figure 5). Cigarette consumption was lower on days 1 and 2 for both groups, which coincides with the requested abstinence period (29.0 ± 6.2 h) between infusion and post-infusion study sessions.

At the post-infusion study sessions there were no significant differences in QSU cigarette craving scores following ketamine (43.6 ± 18.4) and midazolam (44.5 ± 13.8) (Drug Effect: F(1,17) = 0.05, *p* = 0.83, η_p_^2^ = 0.003). Similarly, there were no significant differences in MNWS nicotine withdrawal symptoms following ketamine (8.1 ± 6.1) and midazolam (9.9 ± 8.9) (Drug Effect: F(1,17) = 0.79, *p* = 0.39, η_p_^2^ = 0.044).

### 3.4. Qualitative Feedback

During ketamine infusions, many participants reported having what one participant described as “psychological experiences,” including seeing things, feeling emotional, feeling like they were dreaming, feeling “spacey” and “like time was moving really slowly.” Another patient described it as feeling like they were on a roller coaster. These psychological experiences were reported at high rates (over 70%) by both participants who did and did not correctly identify receiving ketamine. Other common experiences reported by participants were feeling very relaxed and feeling like they did not have any worries. Negative experiences from ketamine infusions included feeling dizzy, which was the most common negative description, and having nausea and vomiting. A few participants said that they felt scared during the infusion. One interesting difference in experiences among participants was that some participants were very sleepy during and following the infusion while others felt like they went about their day normally.

**Figure 6:**
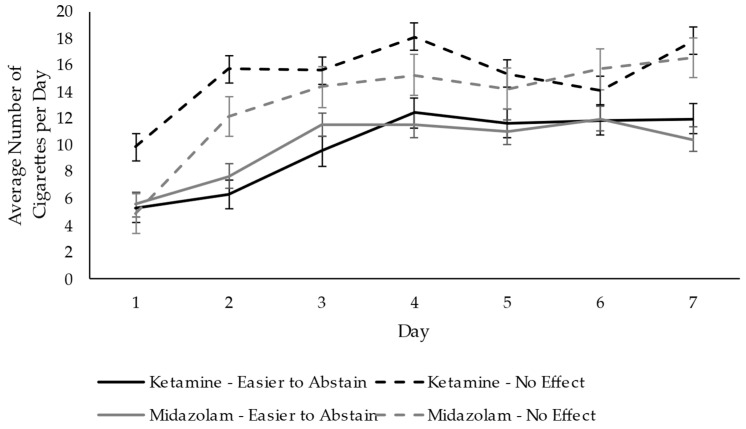
Cigarettes smoked per day following infusion of ketamine and midazolam, stratified by subjective report of an effect on abstaining. Participants were instructed to abstain from smoking between the infusion (day 1) and post-infusion study sessions (days 2–3: 29.0 ± 6.2 h).

While almost half of the participants responded “no” when asked if they had a change in thought patterns, behaviors, or habits following the ketamine infusion, many participants reported decreases in cravings and loss of interest in smoking. Positive experiences reported by a few participants included decreases in anxiety in situations that previously would make them anxious and the ability to focus and not be overwhelmed with tasks needing to be done. One participant described the ketamine infusion as a “life transforming” experience that eliminated intrusive thoughts. Other positive changes included decreases in anxiety, increases in mental clarity, and changes in cigarette taste perception. Some participants who reported decreases in cravings and a better ability to abstain from smoking reported that they felt like these effects wore off either later in the day or within a few days. However, participants who recalled an easier time abstaining after ketamine (*n* = 9, plus 1 participant removed from the quantitative data) reported a similar decrease in cigarettes smoked compared to participants who did not (*n* = 8) during the requested abstinence period (Drug x Group Effect: F(1,15) = 0.001, *p* = 0.9, η_p_^2^ = 0.000). Across the 7-day period, the reported qualitative effect on ease of abstaining had a modest but non-significant effect on the comparison of cigarettes smoked between groups over time (Drug x Time x Qualitative Effect: Β = 1.14, 95% CI: −0.18, 2.47, *p* = 0.09). That is, the rate of increase in cigarette consumption was non-significantly faster after midazolam in the group reporting no benefit of ketamine infusion. However, in the group reporting subjective benefit, overall cigarette consumption was lower following both ketamine and midazolam infusions in comparison to the group reporting no benefit (Figure 6).

During midazolam infusions, the most common experience reported by participants was that “nothing happened” or that they felt normal. Participants also made comments about feeling calm, relaxed, or sleepy. Participants generally reported no changes in their thought patterns, behaviors, habits, or abstinence following the midazolam infusion. See the Supplementary Information for complete details.

## 4. Discussion

In this study, we tested single infusions of ketamine (0.71 mg/kg over 52 min) and the active placebo, midazolam (0.025 mg/kg over 52 min), on safety and tolerability, cigarette smoking, craving and withdrawal symptoms, and qualitative experiences among non-treatment seeking adults with TUD. Both infusions were safe and generally well tolerated. While some expected physical and psychological side effects were noted in both quantitative questionnaires and qualitative interviews, these were mild, transient, and did not require additional medical or psychological follow-up.

In the period of requested cigarette abstinence (between the infusion and post-infusion study sessions), there was a slight, non-significant difference in cigarettes smoked. This difference had a large effect size, although future studies with larger sample sizes are needed to confirm if this is a reproducible effect of ketamine. This slight reduction in cigarette smoking was mirrored by a non-significantly larger decrease in breath CO pre- to post-ketamine infusion compared to midazolam. There were no significant differences in measures of cigarette craving or withdrawal following ketamine and midazolam infusions, which may have been due to the similar number of cigarettes smoked during that period.

Ketamine did not affect the number of cigarettes smoked across the full seven day period following the infusion. This is not unexpected among this non-treatment seeking sample and could suggest that motivation to change behavior (or, in the case of this study, motivation to adhere to a research protocol) may be necessary to realize any potential benefit of ketamine in TUD. Interestingly, although about half of participants reported it was easier to abstain or reduce their cigarette smoking after ketamine during the qualitative interview, these participants did not smoke significantly less after receiving ketamine in comparison to midazolam over the period of requested abstinence or the full 7 days following infusion. This information appears contradictory and could be due to poor retrospective recall of smoking behavior, but it may also reflect momentary changes in perception that were memorable (e.g., not feeling the urge to smoke at an anticipated moment, or not taking pleasure in smoking as typically experienced) without translating to measurable changes in behavior. It is also possible that the effects noted qualitatively were not captured by the quantitative assessment tools used in this study. It is important to note the exploratory nature of the interaction between reported ease of abstinence and cigarette smoking given the small sample size of this study.

Altogether, these results suggest that ketamine, if beneficial for reducing cigarette use, could have greater effects when individuals are actively attempting to abstain from smoking. This differs from previously reported findings of the influence of ketamine on ad lib cocaine use, in which there was a drug effect on reducing use over a period of 3 days despite study participants not actively seeking change [18]. Similarly, in a rat model (presumably without conscious motivation to change behavior), ketamine infusion was found to reduce nicotine self-administration [7]. However, in human trials, currently utilized pharmacologic treatments for tobacco use disorder such as varenicline [29] and bupropion [30] have not demonstrated a benefit in reducing ad lib smoking in subjects not motivated to quit. Therefore, while there is a small amount of evidence to indicate ketamine may be a beneficial intervention in other substance use disorders in pre-contemplative individuals, the results of this study suggest that a conscious motivation to quit or reduce intake in individuals with tobacco use disorder may also be required to realize any potential benefit. This aligns with data from a study of individuals with tobacco and another substance use disorder, over half of whom rated quitting cigarettes as more difficult than their “problem substance” [31].

Drug expectancy may play a role in the therapeutic efficacy of hallucinogens and psychedelics, as well as the personal experience of the drug-induced state, for several reasons. Firstly, individual’s expectations of the drug experience may bias who is willing to volunteer for clinical trials [32], and expectations may be influenced by media coverage. Secondly, it is particularly challenging to maintain an effective blind with a non-hallucinogenic active placebo, although midazolam is considered a superior placebo to saline for ketamine research [33]. Thirdly, individuals who report stronger “mystical” experiences, or personally meaningful experiences, during the drug-induced state may report greater efficacy. For example, among individuals with cocaine use disorder, scores on the Hood Mysticism Scale mediated the effect of ketamine on decreased cocaine use and craving [34]. This could be due to a combination of the molecular actions of the drug as well as a psychological component inspiring motivation for behavioral change.

Despite a robust dissociative effect of the drug, only seven participants correctly identified they had received ketamine following its infusion. Several participants reported a mystical-type experience during ketamine, but on average, scores on the Hood Mysticism Scale were neutral. Our sample of participants had no self-reported prior experience with hallucinogens, and we did not frame the drug infusions as having a potential benefit for smoking cessation. Altogether, while we did not specifically query drug expectancies, this may reflect their lack of prior knowledge about ketamine and suggests low drug expectancy among this sample. The qualitative interview revealed that experiences varied widely, as one participant reported a life-transforming experience while others reported no differences in thoughts, behaviors, or habits. Together with our participants’ lack of interest in quitting smoking, this suggests that this single dose of ketamine had little direct effect on TUD.

The strengths of this study include the within-subject, double-blinded design, and the inclusion of both quantitative and qualitative methods. However, this study has several limitations. Notably, it seems that midazolam did not sufficiently serve as an “active control” for ketamine. Over half of the participants believed midazolam was an inactive placebo, and the study coordinators correctly identified 94% of the midazolam infusions. Differences in psychological experiences between ketamine and midazolam infusion were also noted in quantitative outcomes as well as qualitative interviews. Although neither drug was framed as having the potential to provide benefit for TUD, the risk for bias in reporting outcomes remains a valid concern given this clear difference in experience between the two drugs. While these known differences in the psychological effects of ketamine and midazolam are not entirely dose-dependent, it is possible that midazolam could have served as a more “active” control at a higher dose. The midazolam infusion protocol utilized in the present study was modeled after a similar study in subjects with cocaine use disorder [17]. However, in studies of ketamine’s efficacy in treating major depressive disorder, midazolam has often been dosed at 0.045 mg/kg over 40 min, in contrast to the 0.025 mg/kg over 52 min utilized in this study [35,36,37].

Another limitation was the small sample size, as a larger sample may have revealed more robust trends in measures of smoking habits, withdrawal, and craving following ketamine infusion. Additionally, a large, diverse sample would enable analysis of individual characteristics that may influence the potential to benefit from ketamine infusion in individuals with tobacco use disorder. The lack of a true period of abstinence between infusion and post-infusion study sessions also limits the evaluation of the impact of ketamine infusion on nicotine craving and withdrawal.

Despite a lack of evidence for a direct effect of ketamine on TUD, for some people, ketamine may have a beneficial effect on mood or behavior that could indirectly support a smoking cessation attempt. Sub-anesthetic racemic ketamine is increasingly administered off label for a variety of psychiatric and neurologic conditions such as depression, anxiety, posttraumatic stress disorder, and chronic pain. The safety and efficacy of ketamine for these indications are under investigation, and the dose and administration protocols vary across clinics [38,39]. However, many of these conditions are highly comorbid with TUD, and smoking cessation improves mood symptoms and pain severity [40,41,42]. There is an opportunity to develop holistic ketamine interventions for individuals with TUD and comorbid disorders characterized by anxiety and distress, which commonly trigger the urge to smoke [43,44]. Although we did not specifically recruit participants with anxiety or distress, several participants reported feeling less anxious following the ketamine infusion. This could help support a smoking cessation attempt by alleviating distress-induced craving and perhaps by facilitating change when combined with motivation and/or psychosocial interventions. Additionally, given the relatively invasive and resource-intensive nature of ketamine administration in comparison to currently utilized treatments for tobacco use disorder, future work should also focus on specific populations and circumstances in which its use should be considered if evidence of efficacy is established.

## 5. Conclusions

Among adults with tobacco use disorder who were not interested in changing their smoking behavior, a 0.71 mg/kg infusion of ketamine was well tolerated. However, ketamine appeared to have little to no direct effects on quantitative measures of cigarette smoking, craving, or withdrawal symptoms. Ketamine reportedly reduced qualitative measures of craving and anxiety and about half of participants reported it was easier to abstain or reduce their cigarette smoking after ketamine. Future studies should investigate whether ketamine can indirectly support smoking cessation among individuals with comorbid psychiatric indications for ketamine treatment.

## Figures and Tables

**Table 1:** Demographic and baseline smoking characteristics of the study population. Continuous variables are reported as mean ± standard deviation.

Participant Characteristics	*n* = 18
Age (Years)	45.9 (±13.9)
Sex (Male/Female)	9/9
Race (White/Black/Other)	11/6/1
Cigarettes per Day	15.3 (±7.1)
Pack Years ^1^	21.5 (±16.7)
Baseline Breath CO (ppm)	21.4 (±11.6)
FTND Score ^2^	5.6 (±2.3)
DSM-5 Tobacco Use Disorder Score ^3^	5.1 (±2.5)
E-Cigarette Use (Never/Past/Current)	13/1/4
Highest Degree (None/High School/Technical or Associate’s/Bachelor’s/Advanced Degree)	2/10/4/2/0

^1^ Pack years is the average number of cigarette packs smoked daily multiplied by the number of years smoking. ^2^ FTND = Fagerstrom Test for Nicotine Dependence. ^3^ DSM = Diagnostic and Statistical Manual of Mental Disorders. FTND scores of 3–4 indicate low to moderate nicotine dependence, scores of 5–7 indicate moderate dependence, and scores of 8 or higher indicate high dependence. DSM-5 TUD scores of 2–3 indicate mild severity of TUD, scores of 4–5 indicate moderate severity, and scores of 6 or higher indicate severe TUD.

**Table 2:** Perceptions of study participants and the study coordinator on the drug that was administered during each infusion session, reported as the number of responses for each drug option provided. Both participants and the study coordinator were blinded to the drug that was administered.

	Ketamine		Midazolam	
Drug Believed to Be Administered	Participants	Study Coordinator	Participants	Study Coordinator
Ketamine	7	15	1	1
Midazolam	6	3	1	17
Dexmedetomidine	0	0	1	0
Inactive Placebo	0	0	12	0
Unsure	5	0	3	0

**Figure A1:**
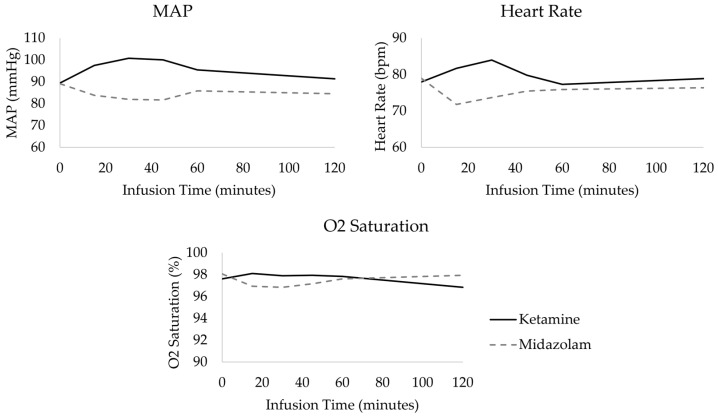
Trends in vital signs (from top left to bottom: mean arterial pressure (MAP), heart rate and oxygenation saturation) during and after infusion of ketamine and midazolam. Infusions lasted 52 min. bpm = beats per minute.

## Data Availability

The quantitative datasets presented in this article are not readily available because the data are part of an ongoing study involving analysis of MRI data. Requests to access the datasets should be directed to the corresponding author. Qualitative data will not be shared to protect the individuals’ privacy.

## Supplementary Materials

The following supporting information can be downloaded at https://www.mdpi.com/article/10.3390/brainsci16050496/s1, Qualitative Feedback Report.

## Abbreviations

The following abbreviations are used in this manuscript:

FDA	Food and Drug Administration
NMDA	N-methyl-D-aspartate
LSD	Lysergic acid diethylamide
TUD	Tobacco use disorder
BDNF	Brain-derived neurotrophic factor
mTOR	Mechanistic target of rapamycin
CO	Carbon monoxide
ppm	Parts per million
DSM	Diagnostic and Statistical Manual of Mental Disorder
MRI	Magnetic resonance imaging
NS	Normal saline
CADSS	Clinician Administered Dissociated States Scale
PANAS	Positive and Negative Affect Schedule
HMS	Hood Mysticism Scale
QSU	Questionnaire of Smoking Urges-Brief
MNWS	Minnesota Nicotine Withdrawal Scale
ANOVA	Analysis of variance
GEE	Generalized Estimating Equation

## Appendix A. Inclusion and Exclusion Criteria

### Appendix A.1. Inclusion Criteria

1. Between the ages of 18 and 65.
2. Smoke ≥ 5 cigarettes/day and agree to abstain from other tobacco/nicotine use during the study period.
3. Smoked ≥ 2 years.
4. Afternoon expired CO concentrations ≥5 ppm or morning urinary cotinine ≥200 ng/mL.
5. Negative urine drug screen for illicit psychotropic drugs (except for THC) and negative breath alcohol concentration.
6. Do not plan to change smoking behavior within the next 60 days.
7. Keep normal daylight hours (awake between 8 am and 9 pm).

### Appendix A.2. Exclusion Criteria

1. Have an unstable medical condition or stable medical condition that would interact with study drug or participation, including chronic pulmonary disease, coronary artery disease, cardiac disease or dysrhythmia, current brain tumor, current increased intracranial pressure or impaired consciousness, or current severe sleep apnea.
2. History of serious head trauma or neurological disorder (e.g., seizure disorder).
3. Have any of the following: hypertension (i.e., systolic ≥140 mm Hg and/or diastolic ≥90 mm Hg on three separate measures; systolic >170 or diastolic > 110 on any occasion.
4. Past or current psychotic disorder (e.g., schizophrenia or bipolar disorder I), current major depressive disorder, or any current psychiatric problems that cause significant distress and interfere with ability to function at home, work, school, or in relationships and/or use of anti-psychotics, lithium, or second-line treatments for depression/anxiety. Psychiatric symptoms that are controlled by first-line treatments for depression/anxiety (e.g., SSRIs) are not exclusionary. Meet DSM-5 criteria for moderate to severe substance use disorder within the past 6 months other than tobacco/nicotine.
5. Daily use of psychotropic medications or other drugs that would interact with study drug (e.g., opioids, methadone, buprenorphine, benzodiazepine, antipsychotics) or taking any psychotropic prescription for <3 months at current dose.
6. History of use of ketamine or benzodiazepines for nonmedical purposes.
7. Among females, pregnancy or lactation.
8. Body Mass Index > 35 and/or weight > 350 pounds.
9. Positive urine drug screen for methamphetamine, cocaine, benzodiazepines, opioids, PCP, or barbiturates.
10. Positive breath alcohol concentration.
11. Vision cannot be corrected to 20/40.

## Appendix B. Vital Sign Trends

On average, ketamine was associated with a slight, transient increase in blood pressure, heart rate, and oxygen saturation, while the opposite was true of midazolam (Figure A1). Mean arterial pressure (Drug x Time: F(5,13) = 9.60, *p* < 0.001, η_p_^2^ = 0.787), heart rate (Drug x Time: F(5,13) = 2.416, *p* = 0.093, η_p_^2^ = 0.482), oxygen saturation (Drug x Time: F(5,13) = 2.485, *p* = 0.086, η_p_^2^ = 0.489).
