# Supplementary material for: Effects of a Single Sub-Anesthetic Dose of Ketamine in Tobacco Use Disorder: An Active-Placebo, Randomized Crossover Study
Source: Brain Sci. 2026 Apr 30;16(5):496. doi: 10.3390/brainsci16050496 (PMC13204344; doi:10.3390/brainsci16050496)
Supplement: Supplementary file 1 [file brainsci-16-00496-s001.zip › brainsci-4215714-supplementary.pdf]

SUPPLEMENTARY INFORMATION

Effects of a Single Sub-Anesthetic Dose of Ketamine in Tobacco Use Disorder: An Active-Placebo, Randomized Crossover Study

Qualitative Feedback Report

Methods

Data Collection: Individual interviews were conducted over the phone by a member of the study team. Each participant was asked three semi-structured questions, with the study team member asking follow-up questions if necessary for clarification. Interviews lasted from 1:48 to 9:07 minutes, with an average length of 4:11 minutes. Interviews were audio-recorded and transcribed via a professional transcription service. Transcripts were then reviewed and compared to the audio files by the study team member to ensure accurate transcription.

Data Analysis: A study team member reviewed the transcripts and developed a codebook to capture concepts found in the data. Both deductive and inductive coding strategies were used to develop the codebook, using concepts from the interview guide and research questions as well as ideas that emerged from the participant discussions.

Interview transcripts were imported and coded in ATLAS.ti version 24 software (Berlin, Germany; Lumivero, LLC). Two researchers independently coded the textual data and met to compare coding. Discrepancies in coding were discussed and resolved iteratively. The codebook was adjusted as needed based on discussions of code meanings and application. Text segments were reviewed by code and summarized. Code summaries were synthesized into themes and organized using principles of thematic analysis [1].

Researchers identified key findings or themes using a combination of the following criteria: (1) prevalence, or the frequency with which an idea is shared across participants; (2) strength, or the emphasis participants give to a point; and (3) valence, or how relevant the point is to the research question or context.[1]

Results:

Ketamine Participant Experiences: When asked about their experiences during the ketamine infusion, many participants reported having what one participant described as "psychological experiences," including seeing things, feeling emotional, feeling like they were dreaming, feeling "spacey" and "like time was moving really slowly." Another participant described it as feeling like they were on a roller coaster.

> *"...the 2nd one, it was like another world. I felt like something off of a cartoon. That's the way I feel, like animated. I feel like everything was animated." (P303)*

"Well, the 1st one it was crazy. I tripped. I thought my hands were rubber and it was...it was crazy. It was great though." (P329)

Other common experiences reported by participants were feeling very relaxed and feeling like they didn't have any worries.

"It was very euphoric for me. I felt like i was floating a little bit, but not laying there with just no cares at all. It seemed like, very relaxing, one of the best buzzes I've ever had." (P332)

"I was so relaxed that I didn't want to speak. But I did not feel bad at all, felt very good." (P314)

Negative experiences from ketamine infusions included feeling dizzy, which was the most common negative description, as well as having nausea and vomiting. Participants also described feeling dizzy and sleepy. A few participants said that they felt scared during the infusion.

"Yeah, the 2nd one. Is the one I felt it on. Felt dizzy, kind of paranoid, like real heavy headed. Dry mouth." (P307)

"Yeah, that was that that was a little scary. I was like, oh, snap like this is, this is really happening." (P312)

"And then of course a lot of nausea, vomiting, not enjoyable experience." (P325)

"...but the 2nd time, I felt it, made me dizzy and upset to my stomach, kinda. I don't really like it at all." (P307)

One interesting difference in experiences among participants was that some participants were very sleepy during and following the infusion while others felt like they just went about their day normally.

"Okay, so my experience on the 1st one um it made me feel really groggy and loopy. Um, um. Yeah, just groggy and loopy, like really sleepy. Um, I was just kind of sleepy the whole entire day. (P308)

"The 1st infusion was a little weird, um, I felt like I was falling in and out of sleep, but I could still hear and I always felt like I knew exactly where people were standing and you know, being around me, and everything, it was just weird I was really drowsy the rest of the day and the next day as well, but then it just kind of wore off and I didn't have any effects. Lasting effects or anything from it was just a weird experience for that few hours. And then I was good." (P305)

"And once it was over, pretty much came back to reality really quickly. And once I got home, I just went on with my day as I regularly did. I went to the grocery store, it seems like I went somewhere else. (P314)

Changes in Thought Patterns, Behaviors and Habits, and Abstinence Following Ketamine: While almost half of the participants responded "no" when asked if they had a change in thought patterns, behaviors, or habits following the ketamine infusion, many participants reported decreases in cravings, increases in abstinence, and other positive changes, including decreases in anxiety, increases in mental clarity, and changes in thought patterns.

> *"...but the 2nd infusion I pretty much lost the taste for smoking. I don't want a cigarette to save money now. Maybe it's horrible actually. I don't know if it was psychological or what, but, um. I smoke a lot later now. Before I was smoking about 17 cigarettes a day. Now it's like 6." (P303)*

> *"The 1st infusion. Yeah, I mean, I had no desire. I was tired, but just the thought of smoking, I was just uninterested in it. Um, for a majority of the time. Um, I don't know, I just didn't have that the urge to smoke." (P305)*

> *"Um. After the 1st infusion, I did notice that I didn't have the a strong urge to smoke as I usually do. [Lasted] about 3 days. It was nice. Actually, yeah, sometimes I wake up in the morning and it's, you know, I have a heavy chest, uh, hard to breathe, but after that infusion, you know, I could tell a difference. Yeah, my chest wasn't as heavy and, you know, the, the urge to smoke as much wasn't there. I really enjoyed that actually." (P313)*

> *"But the second one, it was kind of hard from a vision and maybe because of the medicine or maybe I was too hyper. But it came down to the end. It works. I feel better. The second one, it was kind of hard, because when I first, the second test and the cigarette started tasting kind of funny. I thought it was like kind of stale. Because maybe because the taste in my mouth and the stuff going through me that when I fired up a cigarette when I got through, I couldn't taste it at first. I couldn't taste the nicotine. I said, that's something. It was like stale." (P317)*

Some participants who recognized decreases in cravings and ability to increase abstinence from smoking reported that they felt like the ketamine infusion wore off, either later in the day or within a few days.

> *"But of course, later on, once everything was totally wore off, like I told you how I woke up and that's when I did crave a cigarette. Once I ate, that did it." (P318)*

> *"I quickly went back after 3 days, kind of to my normal routine, but, um, yeah, it did seem easier." (P304)*

Positive experiences reported by a few participants included decreases in anxiety in places that previously would make a participant anxious and the ability to focus and not be overwhelmed with all of the things needed to be done.

"...but after infusion 1 again, those places, physical spaces that would typically make me anxious, I would notice myself, like, actively not having anxiety. I guess I'm so used to having anxiety in a grocery store or Target or something that it stood out to me that I wasn't having those. I would say that was the biggest thought behavior difference." (P304)

"Well, of course, with the first infusion, I would say it was a whole life transforming thing with the intrusive thoughts were out of my mind. I could think more clearly about the current task at hand and the next task at hand instead of being overwhelmed about 10 tasks that needed to be done. I can concentrate on one and then even go and sit down maybe for a little bit before I would go to the next one where usually I wouldn't allow myself to rest when I had those many things that I needed to do." (P314)

Midazolam Participant Experiences: Participants reported not feeling anything or not feeling any changes during the midazolam infusions, with some comments of feeling sleepy, but others saying they didn't feel any sedation at all during the infusion.

"For infusion 2, it wasn't very memorable. I don't really remember much of anything that stands out just because it was a pretty sober experience. I don't, I didn't really even feel sedated. So, pretty unremarkable, but, yeah, between the 2, the personal experience was positive." (P304)

"Nothing, nothing happened at all. I just I didn't feel anything." (P329)

"Um, even afterwards, um. I craved a cigarette really, really bad." (P308)

"Okay, so the first one, because it went so smooth, but I got a little sleepy, I just figured that was the actual infusion. So, I did fall asleep, so I'm not sure what caused me to go to sleep, but I went to sleep." (P318)

"I was calm and it was relaxing." (P336)

Changes in Thought Patterns, Behaviors, Habits, or Changes in Abstaining Following Midazolam: Participants generally reported no changes in their thought patterns, behaviors, habits, or abstinence following the midazolam infusion.

One participant said that it was easier to abstain following midazolam because they only had to wait one day between the infusion and the MRI, whereas they had to wait more days between their infusion and MRI with ketamine. Another participant stated that their cravings were the same, but they abstained from smoking because they wanted to be compliant with the study. There was one participant who was an outlier in daily cigarette use that stated it was easier to abstain following midazolam.

"But the 2nd one, no, it was the same normal mood. I smoked a pack as I usually do, after the 2nd infusion." (P312)

"After infusion 2 it was I definitely had cravings just like usual." (P304)

"After the 2nd infusion, it definitely had had no effect on it." (P304)

Additional thoughts: Participants reported only positive thoughts about their experiences of being in the study and the study staff, especially the study coordinator, with several participants commenting on her making them feel comfortable.

Participants stated that they enjoyed the study, it was easy to do, they felt like there was good reimbursement, the process was safe, and they would recommend it to others.

"Um I think the study is awesome. Really. You know. If you can do infusions to curb your habit of smoking and possibly quit. That's awesome. And I'm all for that." (P313)

"I really enjoyed working with the study coordinator. [They were] absolutely wonderful. And it was a life-changing experience for me and I'm very grateful that I got to do it. I feel very blessed and grateful that I got to do it and got to do it with [them]." (P314)

Limitations: Perspectives in the study were somewhat circumscribed by the small sample size and limited number of interview questions, which may have reduced the depth and variety of viewpoints presented in this analysis. However, the participants in the study provided sufficient amounts of feedback to support and synthesize quantitative data gathered during this intervention.

Reference

1. Braun, V.; Clarke, V. Using thematic analysis in psychology. *Qualitative Research in Psychology.* **2006.** 3(2): 77-101.
